# Vitamin E Regulates the Collagen Contents in the Body Wall of Sea Cucumber (*Apostichopus japonicus*) via Its Antioxidant Effects and the TGF-β/Smads Pathway

**DOI:** 10.3390/antiox13070847

**Published:** 2024-07-15

**Authors:** Zitong Wang, Rujian Xu, Hongbing Yang, Ruixue Li, Jun Ding, Yaqing Chang, Rantao Zuo

**Affiliations:** Key Laboratory of Mariculture and Stock Enhancement in North China’s Sea (Ministry of Agriculture and Rural Affairs), Dalian Ocean University, Dalian 116023, China; 17325222382@163.com (Z.W.); 13955025172@163.com (R.X.); 17635536044@163.com (H.Y.); lrx20010505@163.com (R.L.); dingjun1119@dlou.edu.cn (J.D.); yqchang@dlou.edu.cn (Y.C.)

**Keywords:** *Apostichopus japonicus*, VE, antioxidant, TGF-β/SMAD, collagen

## Abstract

A 70-day feeding experiment was performed to investigate the effects of dietary vitamin E at different addition levels (0, 100, 200, and 400 mg/kg) on the growth, collagen content, antioxidant capacity, and expressions of genes related to the transforming growth factor beta (TGF-β)/Sma- and Mad-related protein (SMAD) signaling pathway in sea cucumbers (*Apostichopus japonicus*). The results showed that the *A. japonicus* in the group with 200 mg/kg vitamin E exhibited significantly higher growth rates, hydroxyproline (Hyp) and type III collagen contents, and superoxide dismutase (SOD) activity, as well as the upregulation of genes related to Tenascin, SMAD1, and TGF-β. Additionally, the *A. japonicus* in the group with 100 mg/kg vitamin E exhibited significantly higher body-wall indexes, denser collagen arrangements, improved texture quality, higher activities of glutathione peroxidase (GSH-Px) and peroxidase (POD), as well as the upregulation of genes related to collagen type I alpha 2 chain (COL1A2), collagen type III alpha 1 chain (COL3A1), and Sp-Smad2/3 (SMAD2/3). In contrast, the *A. japonicus* in the group with 400 mg/kg vitamin E showed a decrease in the growth rates, reduced Hyp contents, increased type I collagen contents, collagen fiber aggregation and a harder texture, along with the downregulation of genes related to the TGF-β/SMAD signaling pathway. Furthermore, the *A. japonicus* in the group with 400 mg/kg exhibited oxidative stress, reflected by the lower activities of SOD, GSH-Px, and POD. These results indicated that *A. japonicus* fed diets with the addition of 100–200 mg/kg vitamin E had improved collagen retention and texture quality by increasing the activities of antioxidant enzymes and the expressions of genes in the TGF-β/SMAD signaling pathway. However, the excessive addition of vitamin E (400 mg/kg) induced oxidative stress, which could increase the collagen degradation and fibrosis and pose a threat to the growth and texture quality of *A. japonicus*.

## 1. Introduction

Sea cucumber (*Apostichopus japonicus*), recognized globally for aquaculture, is celebrated for its high nutritional value and unique medicinal properties [1]. The body wall is the primary edible part of *A. japonicus* [2,3]. Collagen, accounting for 70% of the total protein in the body wall, directly affects the market quality of *A. japonicus*, especially its characteristic glutinous texture [4,5].

Vitamin E is a feed additive with exceptional antioxidant properties [6,7]. Its antioxidant mechanism primarily involves reacting with excess free radicals in the body, blocking free-radical chain reactions, and thereby effectively preventing lipid peroxidation and enhancing the overall antioxidant capacity [8]. Additionally, vitamin E positively affects collagen deposition. It has been shown that the addition of vitamin E significantly increased the collagen contents in the muscles of rabbit (*Oryctolagus cuniculus*) and lamb (*Ovis aries*) [9,10]. The addition of vitamin E at a level of 300–500 mg/kg promoted the content of hydroxyproline (Hyp), an essential component of collagen, in the muscles of grass carp (*Ctenopharyngodon idella*) [11]. Adding 55.61–306.19 mg/kg vitamin E to the diets increased the deposition of collagen in the liver and muscles of the Chinese soft-shelled turtle (*Pelodiscus sinensis*) [12]. It was found that the addition of 250 mg/kg vitamin E promoted the growth, nonspecific immunity, and disease resistance of *A. japonicus* [13]. However, the impact of vitamin E on the collagen content and its underlying mechanisms remains largely unexplored in *A. japonicus*.

The collagen synthesis is regulated by the transforming growth factor beta (TGF-β)/Sma- and Mad-related protein (SMAD) signaling pathway [14]. TGF-β promotes collagen synthesis and deposition by activating the SMAD signaling pathway and increasing the collagen type I alpha 1 chain (COL1A1) gene expression [15]. Long-term inflammation triggers oxidative stress, enhances the expression of TGF-β, and results in the excessive deposition of collagen fibers in the extracellular matrix and the formation of fibrosis [16]. Antioxidants can inhibit tissue fibrosis by suppressing the TGF-β/SMAD pathway [16]. Studies have shown that the combined treatment of vitamin E and pentoxifylline suppressed the expression of fibrosis markers induced by transforming growth factor-beta 1 (TGF-β1) in mice (*Mus musculus*) [17]. Additionally, vitamin E improved renal fibrosis in mice (*Mus musculus*) with unilateral ureteral obstruction by inhibiting the TGF-β/SMAD signaling pathway [18]. The combination of selenium and vitamin E protected against TGF-β1-mediated liver fibrosis by reducing the accumulation of type I collagen in stellate cells [19]. Despite this, some researchers have reported the beneficial effects of vitamin E on the collagen synthesis through the TGF-β signaling pathway. It was shown that vitamin E activated the TGF-β/SMAD pathway and increased the collagen content in human bones [20]. Vitamin E plays a protective and promotive role in the growth and differentiation of dermal papilla stem cells by modulating the TGF-β signaling pathway [21]. Thus, it has been postulated that vitamin E may regulate the TGF-β/SMAD signaling pathway and collagen synthesis in a dose-dependent manner. Furthermore, it could be the different tissues that determine whether vitamin E exerts promoting or inhibitory effects on the collagen synthesis. However, there is still a poor understanding of the mechanisms by which vitamin E regulates the deposition of collagen through the TGF-β/SMAD signaling pathway in the body wall of *A. japonicus*.

Therefore, this study aimed to explore the effects of vitamin E on the collagen synthesis, antioxidant capacity, and expressions of key genes in the TGF-β/SMAD signaling pathway in *A. japonicus*, and to provide some scientific evidence for understanding the effects of vitamin E on the collagen synthesis in the body wall of *A. japonicus* and the relevant mechanisms.

## 2. Materials and Methods

### 2.1. Ethics Statement

*A. japonicus* were captive-bred in a local hatchery room. All experimental procedures on the experimental animals in this study strictly followed the relevant national guidelines of China and Dalian Ocean University.

### 2.2. Experimental Diets and Feeding Experiment

In this experiment, fish meal, fermented soybean meal, and kelp powder were used as the main sources of protein. Based on the designed crude protein and crude fat contents, a basic feed formula was formulated (Table 1). Different concentrations of DL-α-Tocopheryl Acetate (provided by Nanjing Dulai Biotechnology, purity ≥ 96%) were added to the experimental feeds to obtain four different vitamin E content groups: 0 mg/kg, 100 mg/kg, 200 mg/kg, and 400 mg/kg.

DL-α-Tocopheryl Acetate was proportionally added to fish oil, and the solid ingredients that had been finely ground were sieved through an 80-mesh sieve according to the feed formula ratio. Subsequently, they were weighed and added sequentially to the fish oil containing DL-α-Tocopheryl Acetate, thoroughly mixed, and stored sealed. For each feeding, the previous feed was mixed with 30% distilled water and kneaded into spherical solid feed with a diameter of approximately 1.5 cm.

The feeding experiment was conducted at the Key Laboratory of Mariculture, Ministry of Agriculture, Dalian Ocean University. The *A. japonicus* used in the experiment were sourced from Dalian Xinyulong Marine Biotechnology Co., Ltd. (Dalian, China). Prior to the experiment, the sea water was first sand-filtered before it was used for the feeding experiment. The *A. japonicus* were placed in the aquaculture system and pre-cultured for two weeks using basic feed. After 36 h of food deprivation, 180 healthy *A. japonicus* were randomly distributed into 12 square tanks of 30 L each, with 15 individuals per tank. The consistency of the initial total weights of the *A. japonicus* in each group was ensured at approximately 195 ± 1 g. The rearing period was scheduled from November to January, totaling 70 days. During the rearing period, feeding was conducted twice daily at 7 a.m. and 4:30 p.m., with a feeding amount equivalent to 3% of the initial body weight of the *A. japonicus*, adjusted based on their daily food consumption. During the feeding experiment, one-third of the water was replaced daily by using a siphon, and residual feed and feces were cleaned every two days. The rearing environment was maintained under dim-light conditions, with the natural sea water temperature slowly decreasing from 18 to 9 °C, the pH maintained between 7.6 and 8.3, dissolved oxygen levels above 6 mg/L, and the ammonia nitrogen and nitrite concentrations kept below 0.05 mg/L.

### 2.3. Sample Collection

After the conclusion of the feeding experiment, a 48 h fasting period was observed, followed by the measuring and weighing of the *A. japonicus* in each tank to calculate the survival rates, weight gain rates, visceral weight rates, specific growth rates, and body-wall indexes. Subsequently, coelomic fluid was extracted from ten *A. japonicus* individuals in each tank using a syringe. The coelomic fluid was then dissected for supernatant post-centrifugation (3500 rpm, 4 °C, 10 min) and stored at −80 °C for future enzyme activity analysis. *A. japonicus* were dissected by using sterile instruments. For each tank, the viscera and body walls from ten *A. japonicus* individuals were individually separated and weighed to calculate the viscera weight ratio. Then, pieces of the body walls (1 cm × 1 cm) were cut off from the middle backs of ten *A. japonicus* individuals. After that, muscle tissue was stripped from the body walls above. Subsequently, the body walls without muscle tissue of three individuals were cut into 5 mm × 5 mm pieces, which were rinsed with sterile saline and fixed in paraformaldehyde for histological observation. At last, the body walls of seven individuals without muscle tissue and pigment layers were pooled into 1.5 mL RNAase-free tubes (Axygen, Union City, CA, USA) and stored at −80 °C for subsequent gene expression and nutritional composition analyses.

### 2.4. Feed Composition Analysis

The nutritional components of the feeds were analyzed using the AOAC (1995) methods. Here, the relevant methods are simply described as follows: the crude protein content was determined by the Kjeldahl method, multiplying the nitrogen content by 6.25; the crude fat content was determined using the Soxhlet extraction method, with ether as the solvent.

### 2.5. Collagen Content Analysis

The Hyp content was determined using a kit (Art. No. A030-2; Nanjing Jiancheng Bioengineering Institute, Nanjing, China) to treat the samples. The operation followed the manufacturer’s instructions, and an Infinite^®^ Pro 200 microplate reader (Tecan, Männedorf, Switzerland) was used to analyze the samples. The absorbance was measured at a wavelength of 550 nm, and the Hyp concentration was calculated from the standards according to the instructions. The collagen content was estimated by multiplying the Hyp content by 8 (AOAC, 2002).

### 2.6. Texture Quality Analysis

A tool knife was used to cut 1.5 cm × 1.5 cm squares from the middle dorsal parts of the sea cucumber body walls, and scissors were used to trim the sea cucumber papillae. A cylindrical probe with a diameter of 20 mm was used for the test, with a deformation of 50% and a trigger force of 1.5 N. The samples were compressed at a speed of 30 mm/min. (ENS-IPRP texture analyzer, Beijing Yingsheng Hengtai Technology Co., Ltd., Beijing, China)

### 2.7. Enzyme Activity Analysis

The activities of peroxidase (POD), glutathione peroxidase (GSH-Px), and total superoxide dismutase (SOD) in the body walls and coelomic fluid of *A. japonicus* were measured by Nanjing Jiancheng Bioengineering Institute Co., Ltd. The peroxidase activity was measured using the Peroxidase Assay Kit (A084-1-1) [22], the glutathione peroxidase activity was measured using the Glutathione Peroxidase (GSH-Px) Assay Kit (A005-1-1) [23], and the total superoxide dismutase activity was measured using the Total Superoxide Dismutase (T-SOD) Assay Kit (Hydroxylamine method) (A001-1-1) [24]. The specific measurement methods were carried out according to the instructions provided with the kits.

### 2.8. Detailed Microscopic Analysis of Collagen Proteins in Body Wall 

Preparation of paraffin sections: First, the *A. japonicus* body walls were cut into pieces no larger than 2 cm × 2 cm and fixed in a solution of 4% paraformaldehyde fixing solution for 24 h. Next, the Leica ASP200S was used to dehydrate, clarify, and paraffin-embed the samples. Then, the Leica EGH50H + C embedding machine was used to embed the samples into paraffin blocks. Subsequently, the Leica RM2245 rotary microtome was used to cut the paraffin blocks into sections with a thickness of 6 μm. Finally, the Sirius Red (Sirius Red) staining protocol and Van Gieson (VG) staining were performed on two consecutive sections, respectively.

Sirius Red staining: Initially, the sections underwent deparaffinization from paraffin to water, followed by the gentle drying of the surfaces. The sections were then submerged in a Sirius Red staining solution for approximately 8 min. Following the staining, the sections were rapidly dehydrated using three changes of absolute ethanol, with the final change extended to between 30 s and 2 min. This was followed by a brief clearing in butanol for around 10 s. The sections were then placed in two fresh xylene baths for 5 min each to finish the clearing process. They were finally mounted with neutral gum for analysis under a polarized-light microscope.

VG staining: The sections were first deparaffinized and were then placed in two xylene baths for 20 min each. This was followed by two ethanol washes (absolute) for 5 min each and a 5 min immersion in 75% ethanol, concluding with a rinse in tap water. The VG staining solution was prepared by mixing 9 mL of VG solution B with 1 mL of VG solution A. The sections were stained in this mixture for about 1 min, quickly rinsed with water, and then swiftly dehydrated three times using absolute ethanol. Finally, the sections were cleared with xylene and mounted with neutral gum for microscopic examination and image collection and analysis.

### 2.9. Real-Time Quantitative PCR

RNA isolation and cDNA synthesis: Total RNA was extracted from the body walls of *A. japonicus* by using TianGen’s RNA Easy Fast kit (DP451) and FastKing gDNA Dispelling RT SuperMix (KR118) (TianGen, Beijing, China) [25]. The RNA integrity was confirmed via agarose gel electrophoresis by using 50X TAE Buffer (B548101) and Agarose, Regular (A620014) (Sangon Biotech, Shanghai, China) [26]. cDNA synthesis was performed under the following conditions: incubation at 37 °C for 15 min, denaturation at 85 °C for 5 s, and storage of the cDNA at −20 °C.

Primer design: Primers for RT-PCR were devised using NCBI’s Primer BLAST, targeting specific sequences within the coding regions identified in the NCBI database. This rigorous approach yielded eight pairs of primers, including the reference gene primer Cytochrome b (Cytb-R), cited directly from the relevant literature [27] (Table 2).

Quantitative PCR setup and analysis: The qPCR was performed by using the FastKing One Step RT-PCR Kit (KR123) (TianGen, Beijing, China) [28]. The qPCR employed SYBR Green I chemistry in a 20 μL reaction mixture containing 10 μL 2× TransStart^®^ Tip Green qPCR Super Mix, 0.8 μL each of forward and reverse primers (20 μM), 2 μL of cDNA, and 6.4 μL of RNase-free water. PCR cycling consisted of an initial denaturation at 95 °C for 120 s, followed by 45 cycles of 95 °C for 5 s, 95 °C for 10 s for annealing/extension, and concluding with a melting-curve analysis.

Standard curve and dilution: Standard curves for the reference and target genes were established using high-expression samples at an initial concentration of 50 ng/μL, followed by a tenfold dilution series across five points, each replicated three times. The optimal reaction cDNA concentration was determined to be 5 ng/μL, achieved by diluting 50 ng/μL cDNA tenfold with DEPC-treated water.

Relative expression levels were calculated using the 2^−ΔΔCT^ method. This involved computing the ΔCT as the difference between the CT values of the target and reference genes, normalizing the ΔCT to the control group to obtain the ΔΔCT, and determining the fold change as 2^−ΔΔCT^. Results were expressed as means and standard deviations.

### 2.10. Formulas for Calculation


$$\mathrm{Survival}\;\mathrm{rate}\;(\%)=N_{f}/N_{i}\times 100$$

$$\mathrm{Weight}\;\mathrm{growth}\;\mathrm{rate}\;(\%)=\left(\;W_{f}-W_{i}\;\right)/W_{i}\times 100$$

$$\mathrm{Visceral}\;\mathrm{weight}\;\mathrm{ratio}\;(\%)=V_{W}/W\times 100$$

$$\mathrm{Specific}\;\mathrm{growth}\;\mathrm{rate}\;(\%/d)=\left\{\left[\ln\left(W_{f}\right)-\ln\left(W_{i}\right)\right]/T\right\}\times 100$$

$$\mathrm{Body-wall}\;\mathrm{index}\;(\%)=P_{W}/W\times 100$$

In the formula, $N_{f}$, $N_{i}$, $W_{i}$, and $W_{f}$. are the initial number, final number, initial average weight, and final weight of the *A. japonicus* in each tank, respectively; $V_{W}$, $P_{W}$, and W are the wet mass of visceral organs, body-wall index, and whole body; T is the feeding period.

### 2.11. Data Analysis

Data were analyzed using SPSS 22.0 statistical software. Initially, the normality of the data distribution was assessed with the Shapiro–Wilk test, and the homogeneity of the variances was evaluated with Levene’s test. Subsequently, one-way analysis of variance (ANOVA) was employed to determine the significance among the dietary groups. If a significance (*p* < 0.05) was detected, Tukey’s multiple comparisons were used to identify the significant differences between the dietary groups. The collagen areas in the histological images were quantified using image analysis software (Image-Pro Plus 6.0). All data are presented as means ± standard errors (SEs).

## 3. Results

### 3.1. Effects of Vitamin E Addition on Growth Performance of A. japonicus

There were no significant differences in the survival rates of the *A. japonicus* between the different dietary groups (*p* > 0.05). As the vitamin E addition level increased, the weight growth rates and specific growth rates of the *A. japonicus* first increased and then decreased, with the peak values observed in the dietary group with the addition of 200 mg/kg vitamin E. The weight growth rates and specific growth rates of the *A. japonicus* fed diets with 200 mg/kg vitamin E were significantly higher than those of the control group (*p* < 0.05). The visceral weight ratios of the *A. japonicus* fed diets with the addition of 400 mg/kg vitamin E were significantly lower than those in the other dietary groups (*p* < 0.05). The body-wall indexes were significantly elevated in the groups fed diets with the addition of 100 mg/kg, 200 mg/kg, and 400 mg/kg of vitamin E compared to those in the control group (*p* < 0.05) (Table 3).

### 3.2. Effects of Vitamin E Addition on Texture Quality of A. japonicus

As the vitamin E addition level increased, the hardness of the *A. japonicus* body walls gradually increased, reaching its highest value in the 400 mg/kg group, which was significantly higher than that in the control group (*p* < 0.05). As the vitamin E addition level increased, the springiness of the *A. japonicus* body walls initially increased and then decreased. The springiness was the highest in the 100 mg/kg group, which was significantly higher than that of the control group (*p* < 0.05). The gumminess of the *A. japonicus* body walls increased with the addition of vitamin E, reaching its highest value in the 400 mg/kg group, which was significantly higher than that in the control group (*p* < 0.05) (Table 4).

### 3.3. Effects of Vitamin E Addition on Collagen Content in Body Walls of A. japonicus

#### 3.3.1. Body-Wall Hyp and Collagen Contents

**Figure 1:**
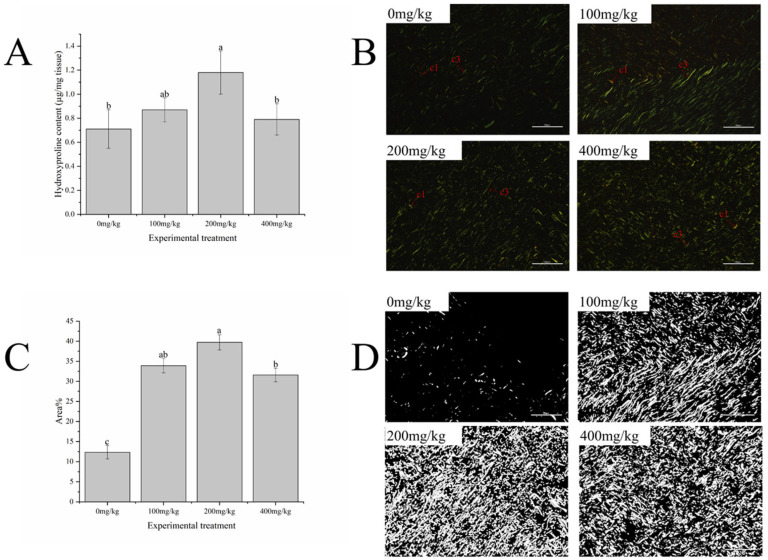
Effects of Vitamin E on collagen contents in the body walls of sea cucumber (*Apostichopus japonicus*). (**A**) The content of hydroxyproline in the body wall; (**B**) Sirius Red staining (200×), c1: type I collagen fibers, c3: type III collagen fibers; (**C**) proportions of collagen in the body walls; (**D**) grayscale image (200×). Note: values are means ± SEs (*n* = 3). Bars bearing different lowercase letters are significantly different between the dietary groups (*p* < 0.05).

As the vitamin E addition level increased, the Hyp contents in the body walls of the *A. japonicus* first increased and then decreased, with the peak values observed in the dietary group with the addition of 200 mg/kg vitamin E. The Hyp contents in the body walls of the *A. japonicus* fed diets with 200 mg/kg vitamin E were significantly higher than those in the control group (*p* < 0.05) (Figure 1A).

#### 3.3.2. Body-Wall Collagen Categories and Distribution

Type I collagen fibers exhibit strong birefringence, with colors ranging from bright orange-red to pale yellow. Type III collagen fibers are densely arranged and have weak birefringence and a green color. In the groups supplemented with vitamin E, both the type I and type III collagen fibers showed significantly higher quantities and denser arrangements than those in the control group. The body walls in the 200 mg/kg group exhibited the most orderly and dense arrangements of collagen fibers, while those in the 400 mg/kg group had the most widespread distributions of type I collagen fibers (Figure 1B).

As the vitamin E addition level increased, the relative area percentages of collagen fibers first increased and then decreased, with the peak value observed in the 200 mg/kg addition group. The relative area percentages of collagen fibers in the 200 mg/kg group were significantly higher than those in the control group and 400 mg/kg group (*p* < 0.05) (Figure 1C,D).

**Figure 2:**
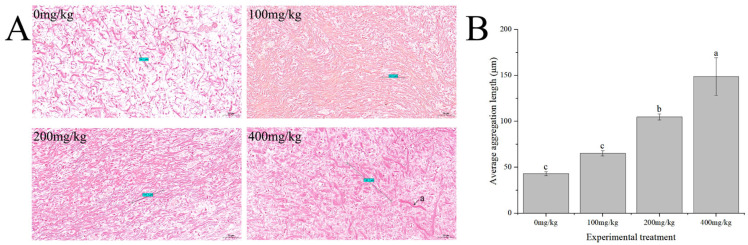
Effects of vitamin E on collagen fiber morphology in the body walls of sea cucumber (*Apostichopus japonicus*). (**A**) Van Gieson staining (200×), a: fiber bundle aggregation; (**B**) average aggregation length (μm). Note: values are means ± SEs (*n* = 3). Bars bearing different lowercase letters are significantly different between the dietary groups (*p* < 0.05).

The pink fibers refer to collagen fibers. In the groups supplemented with vitamin E, the collagen fibers showed significantly higher quantities and denser arrangements compared to those of the control group. The arrangements of fibers in the 100 mg/kg and 200 mg/kg groups were more orderly than those in the control group and 400 mg/kg additive group. As the vitamin E addition level increased, the lengths of the collagen fibers gradually increased, leading to the aggregation of fiber bundles, reaching their maximum values in the 400 mg/kg group (Figure 2).

### 3.4. Effects of Vitamin E Addition on Antioxidant Enzyme Activities of A. japonicus

#### 3.4.1. Antioxidant Enzyme Activities in the Coelomic Fluid of *A. japonicus*

As the vitamin E addition level increased, the SOD, GSH-Px, and POD activities in the coelomic fluid of the *A. japonicus* showed a trend of first increasing and then decreasing. Specifically, SOD reached its highest activity in the group with the 200 mg/kg addition, which was significantly higher than that in the control group (*p* < 0.05). GSH-Px and POD reached their highest activities in the group with the 100 mg/kg addition, which were higher than those in the control group (*p* > 0.05). However, the POD activity in the 400 mg/kg group was lower than that in the control group (Table 5).

#### 3.4.2. Antioxidant Enzyme Activities in the Body Walls of *A. japonicus*

As the vitamin E addition level increased, the SOD and POD activities in the body walls of the *A. japonicus* showed a trend of first increasing and then decreasing. Specifically, SOD reached its highest activity in the group with the 200 mg/kg addition, which was significantly higher than that in the control group (*p* < 0.05). GSH-Px reached its highest activity in the group with the 400 mg/kg addition, which was higher than that in the control group (*p* > 0.05). POD reached its highest activity in the group with the 100 mg/kg addition, which was higher than that in the control group (*p* > 0.05). However, the POD activity in the 400 mg/kg group was lower than that in the control group (Table 6).

### 3.5. Effects of Vitamin E Addition on Expressions of Collagen and TGF-β/Smads Pathway-Related Genes in Body Walls of A. japonicus

**Figure 3:**
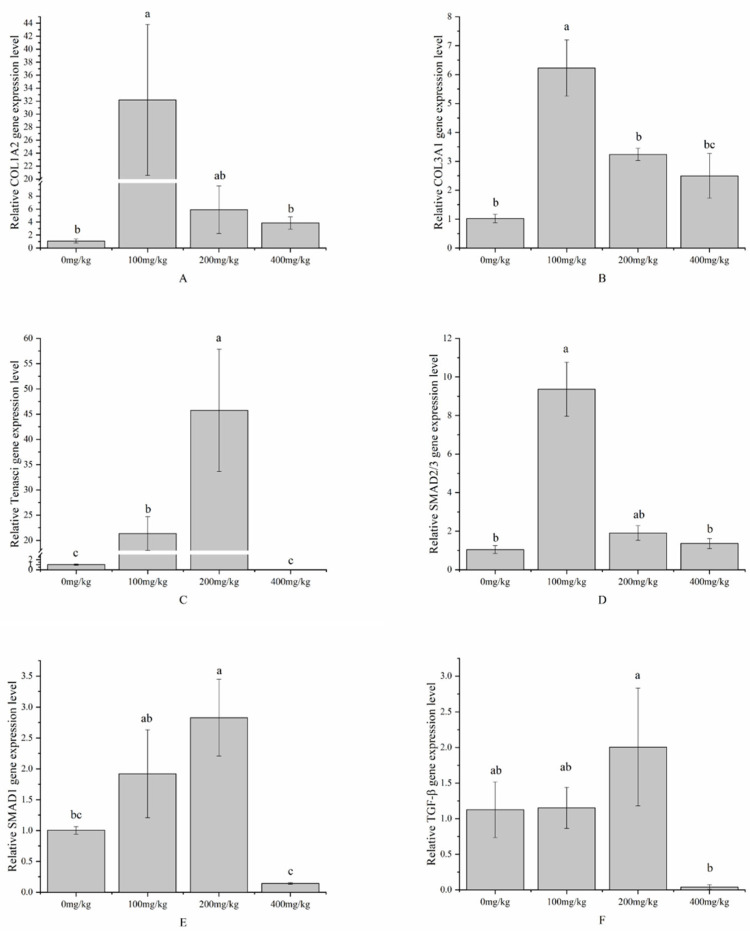
Effects of different vitamin E addition levels on collagen and TGF-β/SMAD pathway gene expression in the body walls of sea cucumber (*Apostichopus japonicus*). Note: values are means ± SEs (*n* = 3). Bars bearing different lowercase letters are significantly different between the dietary groups (*p* < 0.05). (**A**) Col1A2; (**B**) Col3A1; (**C**) Tenascin; (**D**) SMAD2/3; (**E**) SMAD1; (**F**) TGF-β.

As the level of the vitamin E addition increased, the expression levels of the collagen type I alpha 2 chain (COL1A2), collagen type III alpha 1 chain (COL3A1), Sp-Smad2/3 (SMAD2/3), Tenascin, SMAD1, and TGF-β genes in the body walls of the *A. japonicus* showed a trend of first increasing and then decreasing. The expression levels of COL1A2, COL3A1, and SMAD2/3 in the groups supplemented with vitamin E were consistently observed to be higher than those in the control group. Specifically, COL1A2, COL3A1, and SMAD2/3 reached their highest expression levels in the group with the 100 mg/kg addition, which were significantly higher than those in the control group and the group supplemented with 400 mg/kg vitamin E (*p* > 0.05). Tenascin, SMAD1, and TGF-β reached their highest expression levels in the group with the 200 mg/kg vitamin E addition, significantly outperforming both the control and 400 mg/kg groups (*p* > 0.05). However, the expression levels of Tenascin, SMAD1, and TGF-β in the 400 mg/kg group were lower than those in the control group (Figure 3).

## 4. Discussion

Vitamin E is an essential micronutrient for many animal species [7,29]. Although there are many reports on the positive effects of vitamin E on the growth, development, immune regulation, and collagen accumulation in livestock and aquatic animals, research on its application in *A. japonicus* aquaculture is limited. It has been shown that the addition of 0.3 mg/kg of selenium and 200 mg/kg vitamin E to the feed of *A. japonicus* significantly increased the specific growth rate to 1.39 and the weight gain rate to 10.39 with an initial weight of 4.48 g. Additionally, the amylase activity of the *A. japonicus* was significantly increased by 200 mg/kg vitamin E [30]. The addition of 100 mg/kg vitamin E to the feed of *A. japonicus* with an initial weight of 7.96 g has been shown to significantly enhance its specific growth rate and survival rate [31]. In this study, the *A. japonicus* in all the groups showed obvious weight gain and no mortality, indicating that the experimental feeds were conducive to *A. japonicus* survival and growth. The specific growth rates of the *A. japonicus* were effectively improved by the addition of 100 mg/kg and 200 mg/kg vitamin E. Research indicates that the appropriate addition of 80 mg/kg vitamin E to feed can enhance the specific growth rate and feed conversion efficiency of juvenile discus fish (*Symphysodon haraldi*). However, the excessive addition of vitamin E resulted in decreased specific growth rates and digestive enzyme activities [32]. Vitamin E can affect cell growth, differentiation, and functioning by regulating cell signaling pathways, thereby indirectly influencing the absorption function of the intestine [33]. Additionally, vitamin E can improve the feed conversion efficiencies and growth rates of aquatic animals [34]. Moreover, the growth rates of the *A. japonicus* in this study were consistent with the relative areas of collagen fibers in their body walls. This indicated that collagen deposition is critical for body-wall formation and subsequent growth. Thus, understanding the effects of vitamin E on collagen synthesis will be helpful to better elucidate the mechanisms whereby vitamin E regulates the growth performance of *A. japonicus*.

The body wall is the main edible part of *A. japonicus*. The texture characteristics of the body wall are closely related to the collagen content and structure of *A. japonicus* [35]. Hyp is often used to estimate the total collagen, as it constitutes a significant portion of collagen’s unique triple-helical structure [36]. The existing research indicates that feeding hybrid grouper (*Epinephelus fuscoguttatus*) 110 mg/kg vitamin E promoted their growth performance by enhancing the synthesis of Hyp [37]. In this study, the addition of 100 mg/kg, 200 mg/kg, and 400 mg/kg vitamin E showed higher Hyp contents than the control group, with the 200 mg/kg group reaching the highest value (1.18 µg/mg tissue). This could be due to the promoting effects of vitamin E on the Hyp synthesis. Research has shown that TGF-β1 is a key initiator of collagen deposition [16]. The TGF-β/SMAD signaling pathway plays a crucial role in the deposition of collagen in aquatic life [38,39]. In addition, in rainbow trout (*Oncorhynchus mykiss*), cardiac fibroblasts are stimulated by TGF-β1 to synthesize more collagen [40]. In this study, the expression levels of collagen and genes related to the TGF-β/SMAD signaling pathway were the highest in the 100 mg/kg and 200 mg/kg vitamin E supplementation groups. Specifically, adding 100 mg/kg vitamin E resulted in the highest expression levels of COL1A2, COL3A1, and SMAD2/3, while adding 200 mg/kg vitamin E led to the highest expression levels of Tenascin, SMAD1, and TGF-β. This indicates that a balance in the regulation of the collagen synthesis pathway was achieved at the 100 mg/kg and 200 mg/kg vitamin E supplementation levels. Notably, the TGF-β/SMADS signaling pathway was fully activated at the 200 mg/kg vitamin E supplementation level. Furthermore, the collagen content and growth reached their peaks at this level, further supporting this hypothesis.

In this study, Sirius Red staining and polarized-light scanning were used to observe the type I and type III collagen fibers in the body walls of *A. japonicus*. Type I collagen fibers are usually present in the form of thick bundles that can withstand significant mechanical stress and tension. When the content of type I collagen fibers is high, the body wall becomes harder and more resistant to deformation, but softer and less elastic. Type III collagen fibers, being finer, are more flexible and easier to bend, contributing to the elasticity and recovery capacity of the tissues. Therefore, the proportion of type I and type III collagen fibers could affect the texture characteristics of the body wall of *A. japonicus*. Hardness, springiness, cohesiveness, and chewiness are important indicators for evaluating the textural characteristics of food [35]. In this study, the body walls of *A. japonicus* in the groups supplemented with vitamin E (100–400 mg/kg) showed significantly increased quantities of type III collagen fibers and improved springiness and chewiness compared to the control group. Studies have shown that springiness and chewiness are positively correlated with the soft, waxy texture of the body wall of *A. japonicus* [41].

In this study, the groups supplemented with 100 mg/kg and 200 mg/kg vitamin E exhibited more orderly arrangements and increased densities of collagen fibers. This was consistent with the results of a previous study that has shown that vitamin E can enhance the mechanical strength and elasticity of human skin by improving the arrangement and structure of collagen fibers [42]. In the periodontal ligament, the resistance to deformation is directly proportional to the collagen fiber content [43]. Additionally, in bone tissue, the mechanical strength and resistance to deformation are higher when the collagen fibers are more tightly and more orderly arranged [44]. Thus, the body walls of *A. japonicus* fed diets with the addition of 100–200 mg/kg vitamin E will be more resistant to deformation. Furthermore, the body walls of *A. japonicus* in the group with the addition of 400 mg/kg vitamin E exhibited higher hardness and quantities of type I collagen fibers compared to the other groups. The VG staining revealed that the collagen fibers formed thicker and longer fiber bundle aggregations in the 400 mg/kg group. This may be due to the substantial extracellular matrix deposition caused by oxidative stress, hardening the tissue and resulting in fibrosis [45]. At the 400 mg/kg dosage, the expressions of TGF-β and other genes did not increase further, likely due to the oxidative stress in the *A. japonicus* caused by the excessive vitamin E. Vitamin E inhibits the overexpression of the TGF-β/SMADS signaling pathway induced by oxidative stress, preventing its expression [46].

The beneficial effects of vitamin E on collagen deposition could be due to its protective effects on Hyp degradation through its antioxidant properties. Reactive oxygen species (ROS) could participate in the degradation of collagen [47]. It was found that the degradation of Hyp in fish can be reduced by the antioxidant properties of vitamin E [48]. Vitamin E, recognized for its potent antioxidant properties and capacity to neutralize free radicals, plays a crucial role in preventing lipid peroxidation and maintaining the integrity of cellular membranes [49]. The antioxidant enzymes SOD, GSH-Px, and POD within the sea cucumber’s body wall and coelomic fluid primarily function to eliminate harmful free radicals and peroxides, safeguarding cells from oxidative damage and maintaining internal equilibrium and health [50]. In this study, vitamin E at an appropriate supplementation level increased the activities of SOD, GSH-Px, and POD in the *A. japonicus*. However, in this study, the *A. japonicus* fed diets with the addition of 400 mg/kg vitamin E showed decreased antioxidant enzyme activities, which could be due to the induced oxidative stress caused by the excessive vitamin E. This was consistent with the findings of some previous studies [51,52]. Thus, it could be that the increased degradation of collagen accounted for the lower collagen contents in the control and 400 mg/kg vitamin E groups. Furthermore, oxidative stress can also induce the overexpression of the TGF-β1 signaling pathway and thereby promote the transcription of type I procollagen [18]. As mentioned above, the body walls of the *A. japonicus* fed diets with the addition of 400 mg/kg vitamin E exhibited higher quantities of type I collagen fibers compared to the other groups. Despite the suppression of the TGF-β/SMAD signaling pathway-related gene expression in the highest-dose group, the expression levels of COL1A2 and COL3A1 remained higher than those in the control group. This suggests that the antioxidant effect of vitamin E may continue to reduce the collagen degradation through other mechanisms. An appropriate amount of vitamin E can activate the TGF-β/SMAD signaling pathway, thereby promoting collagen synthesis. Although excessive vitamin E can induce oxidative stress, it does not lead to the overexpression of the TGF-β/SMAD signaling pathway, thereby preventing the formation of tissue fibrosis.

Consumer demands for nutritional value and health benefits have led to ongoing enhancements in farming techniques to improve the quality of aquaculture products [53,54]. *A. japonicus*, renowned for their high nutritional and unique medicinal value, hold a prestigious place among aquatic products. Improving their quality is crucial for their market competitiveness and consumer satisfaction [55]. In particular, the body wall thickness is a key indicator of the quality of *A. japonicus*, as it is closely related to the collagen content [56]. From a nutritional perspective, vitamin E promotes the synthesis of collagen, which enhances the body-wall thickness and thereby improves the nutritional value of *A. japonicus* [57]. Additionally, increasing the collagen content enhances the thickness and chewiness of the body wall, thereby improving its market value [58]. This results in the production of higher-quality, more nutritious *A. japonicus*, which benefits consumer health [54]. As a potent antioxidant, vitamin E can protect the cells from the hazards of oxidative stress and thereby significantly improve the health of *A. japonicus* [13]. It is known that the body wall is the primary edible part of *A. japonicus* [2,3]. Its market price is influenced not only by the quality but also by the body-wall index. Vitamin E is known to enhance the body-wall index, thereby increasing the profitability of *A. japonicus* [59,60]. In summary, optimizing the vitamin E levels in the feeds of *A. japonicus* can improve their nutritional value, physiological health, and commercial viability.

## 5. Conclusions

In conclusion, the addition of 200 mg/kg vitamin E to *A. japonicus* feed was shown to activate the TGF-β/SMAD signaling pathway, enhance the growth performance, and promote Hyp synthesis. Additionally, 200 mg/kg vitamin E promoted the synthesis of type III collagen fibers, increased the antioxidant enzyme activities, improved the collagen deposition, and increased the tissue structure and collagen fiber density, thereby improving the texture quality of the *A. japonicus*. However, the addition of 400 mg/kg vitamin E triggered oxidative stress, impaired the growth performance, induced collagen fiber aggregation, and hardened the texture of the body walls of the *A. japonicus*, which could be accomplished by downregulating the TGF-β/SMAD signaling pathway. This study provides valuable insights into improving the nutritional and sensory qualities reflected by the collagen contents by optimizing the addition of antioxidants to the feeds of *A. japonicus.* Further studies are needed to explore the effects of other antioxidants, as well as the interactive effects of different antioxidants, on the collagen synthesis in *A. japonicus*.

## Figures and Tables

**Table 1:** Ingredients and proximate analysis of the experimental diets (g/kg).

Ingredient	Dietary Vitamin E Levels (mg/kg)			
	0 mg/kg	100 mg/kg	200 mg/kg	400 mg/kg
Fish meal ^1^	40	40	40	40
Fermented soybean meal ^2^	50	50	50	50
Sargassum thunbergii meal ^3^	290	290	290	290
Fish oil	10	10	10	10
Vitamin premix ^4^	5	5	5	5
DL-α-Tocopherol Acetate (97% purity)	-	0.1	0.2	0.4
Mineral premix ^5^	5	5	5	5
Sea mud	600	599.9	599.8	599.6
Proximate composition				
Crude protein (%)	8.48	8.51	8.39	8.41
Crude lipid (%)	0.53	0.57	0.55	0.58

Note: ^1^ Fish meal: crude protein, 68.7% dry matter; crude lipid, 2.6% dry matter. Dalian Xinyulong Marine Biological Seed Technology Co., Ltd. (Dalian, China). ^2^ Fermented soybean meal: crude protein, 51.56% dry matter; crude lipid, 0.9% dry matter. Dalian Xinyulong Marine Biological Seed Technology Co., Ltd. (Dalian, China). ^3^ Sargassum thunbergii meal: crude protein, 16.8% dry matter. Dalian Xinyulong Marine Biological Seed Technology Co., Ltd. (Dalian, China). ^4^ Vitamin premix (mg or g kg^−1^ diet): vitamin D, 5 mg; vitamin K, 10 mg; vitamin B12, 10 mg; vitamin B6, 20 mg; folic acid, 20 mg; vitamin B1, 25 mg; vitamin A, 32 mg; vitamin B2, 45 mg; pantothenic acid, 60 mg; biotin, 60 mg; niacin acid, 200 mg; inositol, 800 mg; ascorbic acid, 2000 mg; microcrystalline cellulose, 16.71 g. ^5^ Mineral premix (mg or g kg^−1^ diet): CuSO_4_·5H_2_O, 10 mg; Na_2_SeO_3_ (1%), 25 mg; ZnSO_4_·H_2_O, 50 mg; CoCl_2_·6H_2_O (1%), 50 mg; MnSO_4_·H_2_O, 60 mg; FeSO_4_·H_2_O, 80 mg; Ca(IO_3_)_2_, 180 mg; MgSO_4_·7H_2_O, 1200 mg; zeolite, 18.35 g.

**Table 2:** Real-time PCR primers used in the present study.

Gene	Gene ID	Annealing Temperature (°C)	Primer Sequences (5′-3′)
Cytb-R ^1^	KP170618.1	58	F:5′-TGAGCCGCAACAGTAATC-3′
			R:5′-AAGGGAAAAGGAAGTGAAAG-3′
Tenascin ^2^	c64081.graph_c1	51	F:5′-CCCTGATGGTGCTCT-3′
			R:5′-GGGACGAATCTTCATTTCTGTA-3′
Col1A2 ^3^	c54738.graph_c0	56	F:5′-CGGACTTTTACTTTGGCGTTAT-3′
			R:5′-TTTCTGGCGGTCTGCCTAT-3′
Col3A1 ^4^	BSL78_04953	59	F:5′-GGTCCTTCTTCATCTCTTTGGTCAC-3′
			R:5′-GCTGCCAATGGTACGGTTACAC-3′
SMAD2/3 ^5^	BSL78_11878	59	F:5′-AGAACCACCACGAACTCAAACATG-3′
			R:5′-GCAGACAGCAGCAGGGATAAAC-3′
SMAD1 ^6^	BSL78_15508	58	F:5′-GGCATACTCGCAGCAGTCTAAAG-3′
			R:5′-AGGTGTCTCATCGGAAAGGTCTAC-3′
TGFBR2 ^7^	BSL78_06251	56	F:5′-TTCCTCGTTAGACCATTCGTTGAAC-3′
			R:5′-ATACAATAACAGAGTGCTGGGATGAAG-3′
TGF-β ^8^	BSL78_20284	59	F:5′-ACCGCTCCTCACCCTTTAACAC-3′
			R:5′-CCACTAGCACTAAGCAGCATATCAG-3′

Note: ^1^ Cytb-R, cytochrome b reductase. ^2^ Tenascin, tenascin-c. ^3^ Col1A2, collagen type I alpha 2 chain. ^4^ Col3A1, collagen type III alpha 1 chain. ^5^ SMAD2/3, sp-smad2/3. ^6^ SMAD1, Sma- and Mad-related protein 1. ^7^ TGFBR, transforming growth factor beta receptor 2. ^8^ TGF-β, transforming growth factor beta.

**Table 3:** Effects of vitamin E addition levels on the growth performance of sea cucumber (*Apostichopus japonicus*).

	Dietary Vitamin E Levels (mg/kg)			
	0 mg/kg	100 mg/kg	200 mg/kg	400 mg/kg
Survival Rate (%)	100	100	100	100
Weight Growth Rate (%)	75.19 ± 0.08 ^b^	109.52 ± 0.05 ^ab^	114 ± 0.03 ^a^	106.18 ± 0.10 ^ab^
Specific Growth Rate (%/d)	0.71 ± 0.10 ^b^	0.94 ± 0.07 ^ab^	1.06 ± 0.04 ^a^	0.90 ± 0.04 ^ab^
Viscera Weight Ratio (%)	17.84 ± 0.20 ^a^	12.47 ± 0.11 ^b^	10.69 ± 0.13 ^bc^	6.96 ± 0.01 ^c^
Body-Wall Index (%)	58.77 ± 0.01 ^b^	64.16 ± 0.01 ^a^	62.81 ± 0.02 ^a^	62.02 ± 0.01 ^a^

Note: Values are means ± SEs (*n* = 3). Different superscript lower-case letters within each row represent significant differences (*p* < 0.05). This rule applies throughout.

**Table 4:** Effects of vitamin E (VE) addition levels on the texture profile analysis of sea cucumber (*Apostichopus japonicus*) body walls.

	Dietary Vitamin E Levels (mg/kg)			
	0 mg/kg	100 mg/kg	200 mg/kg	400 mg/kg
Hardness ^1^	42.5 ± 2.1 ^b^	55.81 ± 7.8 ^ab^	62.53 ± 5.7 ^ab^	69.13 ± 10.8 ^a^
Springiness ^2^	1.24 ± 0.6 ^b^	1.60 ± 0.9 ^a^	1.45 ± 0.9 ^ab^	1.50 ± 0.4 ^a^
Cohesiveness ^3^	0.75 ± 0.2	0.73 ± 0.4	0.67 ± 0.5	0.69 ± 0.4
Chewiness ^4^	0.76 ± 0.1	0.91 ± 0.1	0.77 ± 0.1	0.81 ± 0.1
Gumminess ^5^	31.84 ± 1.89 ^b^	39.63 ± 4.51 ^ab^	41.14 ± 3.19 ^ab^	46.12 ± 4.69 ^a^

Note: ^1^ Hardness (N) was defined as the maximum peak value when compressing the sample for the first time. ^2^ Springiness (mm) was defined as the degree to which the sample can be recovered after the first compression. ^3^ Cohesiveness (ratio) was defined as the adhesion inside the sample. ^4^ Chewiness (mJ) was defined as the work required to chew a solid sample (springiness × gumminess). ^5^ Gumminess (N) was defined as the viscosity characteristics of semi-solid samples (hardness × cohesiveness). Values are means ± SEs (*n* = 3). Different superscript lower-case letters within each row represent significant differences (*p* < 0.05). This rule applies throughout.

**Table 5:** Effects of VE addition level on antioxidant indexes of sea cucumber (*Apostichopus japonicus*) coelomic fluid.

	Dietary Vitamin E Levels (mg/kg)			
	0 mg/kg	100 mg/kg	200 mg/kg	400 mg/kg
SOD ^1^ (U/mL)	147.49 ± 0.91 ^b^	153.26 ± 4.63 ^ab^	158.42 ± 1.60 ^a^	157.89 ± 1.62 ^a^
GSH-Px ^2^ (U/mL)	10.72 ± 1.09	13.19 ± 0.82	11.13 ± 0.71	10.80 ± 1.01
POD ^3^ (U/mL)	0.92 ± 0.14	1.27 ± 0.19	1.00 ± 0.11	0.82 ± 0.03

Note: ^1^ SOD, superoxide dismutase. ^2^ GSH-Px, glutathione peroxidase. ^3^ POD, peroxidase. Values are means ± SEs (*n* = 3). Different superscript lower-case letters within each row represent significant differences (*p* < 0.05).

**Table 6:** Effects of VE addition levels on antioxidant indexes of sea cucumber (*Apostichopus japonicus*) body walls.

	Dietary Vitamin E Levels (mg/kg)			
	0 mg/kg	100 mg/kg	200 mg/kg	400 mg/kg
SOD ^1^ (U/mL)	600.05 ± 15.42 ^c^	695.97 ± 12.74 ^ab^	704.63 ± 24.87 ^a^	633.92 ± 25.36 ^bc^
GSH-Px ^2^ (U/mL)	21.51 ± 2.54 ^b^	40.39 ± 2.96 ^a^	33.57 ± 4.49 ^ab^	45.41 ± 8.04 ^a^
POD ^3^ (U/mL)	9.54 ± 0.58	11.96 ± 1.05	10.17 ± 0.69	9.09 ± 1.61

Note: ^1^ SOD, superoxide dismutase. ^2^ GSH-Px, glutathione peroxidase. ^3^ POD, peroxidase. Values are means ± SEs (*n* = 3). Different superscript lower-case letters within each row represent significant differences (*p* < 0.05).

## Data Availability

All relevant data are presented within the paper.
